# Border Control for Infectious Respiratory Disease Pandemics: A Modelling Study for H1N1 and Four Strains of SARS-CoV-2

**DOI:** 10.3390/v15040978

**Published:** 2023-04-16

**Authors:** Nigel Wei-Han Lim, Jue Tao Lim, Borame Lee Dickens

**Affiliations:** 1 Saw Swee Hock School of Public Health, National University of Singapore 12 Science Drive 2, #10-01, Singapore 117549, Singapore; nigellwh@nus.edu.sg (N.W.-H.L.); ephdbsl@nus.edu.sg (B.L.D.); 2 Lee Kong Chian School of Medicine, Nanyang Technological University Experimental Medicine Building, 59 Nanyang Drive, Singapore 636921, Singapore

**Keywords:** diagnosis, quarantine, virus, viral load, transmission, risk

## Abstract

Post-pandemic economic recovery relies on border control for safe cross-border movement. Following the COVID-19 pandemic, we investigate whether effective strategies generalize across diseases and variants. For four SARS-CoV-2 variants and influenza A-H1N1, we simulated 21 strategy families of varying test types and frequencies, quantifying expected transmission risk, relative to no control, by strategy family and quarantine length. We also determined minimum quarantine lengths to suppress relative risk below given thresholds. SARS-CoV-2 variants showed similar relative risk across strategy families and quarantine lengths, with at most 2 days’ between-variant difference in minimum quarantine lengths. ART-based and PCR-based strategies showed comparable effectiveness, with regular testing strategies requiring at most 9 days. For influenza A-H1N1, ART-based strategies were ineffective. Daily ART testing reduced relative risk only 9% faster than without regular testing. PCR-based strategies were moderately effective, with daily PCR (0-day delay) testing requiring 16 days for the second-most stringent threshold. Viruses with high typical viral loads and low transmission risk given low viral loads, such as SARS-CoV-2, are effectively controlled with moderate-sensitivity tests (ARTs) and modest quarantine periods. Viruses with low typical viral loads and substantial transmission risk at low viral loads, such as influenza A-H1N1, require high-sensitivity tests (PCR) and longer quarantine periods.

## 1. Introduction

The COVID-19 pandemic has posed challenging trade-offs for policymakers worldwide, including the minimisation of disease transmission while maximising economic recovery. In particular, the travel and tourism industries have been devastated, losing an estimated $2.1 trillion over 2020–2021 [1]. The easing of travel restrictions is often tentative, as policymakers remove protective measures iteratively to prevent large uncontrolled outbreaks [2].

Many governments were delayed either in their border control responses or their subsequent relaxation [3]. Early in the pandemic, governments implemented full or partial travel bans in the absence of effective vaccines and significant natural immunity [4]. With the introduction of high-efficacy mRNA vaccines reducing the risks of transmission and severe illness [5], countries began national vaccination campaigns and transitions to controlled endemic states [6,7].

The 2009 influenza A-H1N1 pandemic presented similar challenges, originating in the United States and spreading worldwide. The pandemic strain of influenza A-H1N1 differed from seasonal strains in its increased fatality rate and incidence among younger people [8]. This highlights the potential emergence of further variants of concern and merits comparison with SARS-CoV-2 in terms of the transmissibility and effectiveness of border control strategies.

Countries must determine strategies that adequately limit transmission from infected travellers into local communities without being prohibitively costly or disincentivizing travel. Due to global heterogeneity in COVID-19 vaccine coverage [9] and in base incidence rates of disease [10], appropriate quarantine and testing measures likely vary by source country. Furthermore, the generalizability of effective measures across diseases and variants is of interest, as new diseases or variants of concern will likely emerge [11]. Informed decision making can minimise illness spread early in future outbreaks, when data on infectiousness may be especially scarce.

The relative risk of transmission of a given disease or variant under given quarantine and testing measures, compared to the baseline of no measures, is useful in quantifying the measures’ effectiveness. Further, the minimal quarantine length required to suppress this relative risk below given thresholds for each testing regime can quantify the comparative efficacy of testing strategies.

Previous mechanistic models of cross-border SARS-CoV-2 transmission have focused on predicting secondary cases generated by infected travellers under border testing and quarantine measures [12,13]. We refine this work to predict the relative transmission risk (TR) posed by incoming infected travellers under given measures, allowing differing viral loads between individuals and over time. Various simplifying assumptions are relaxed, such as constant TR across infected travellers’ time outside quarantine and a single representative viral load profile applied to all infected travellers. We apply the model to different SARS-CoV-2 variants of concern, and additionally to influenza A-H1N1, to investigate how strategies’ effectiveness varies with viral load kinetics and available tests’ sensitivity profiles.

## 2. Materials and Methods

This study uses Monte Carlo simulation to estimate the probability of missing infected travellers, for given cross-border policies and virus variants. It estimates TR depending on the viral load profile of each such missed traveller, which is used to compare the performance of respective cross-border policies in minimizing the secondary cases generated due to imported cases. No experiments were performed in this study.

Alongside influenza A-H1N1, five variants (here we differentiate between infections with and without vaccination) of SARS-CoV-2 were modelled: prealpha, alpha, delta (unvaccinated), delta (vaccinated) and omicron (vaccinated).

**Scheme 1:**
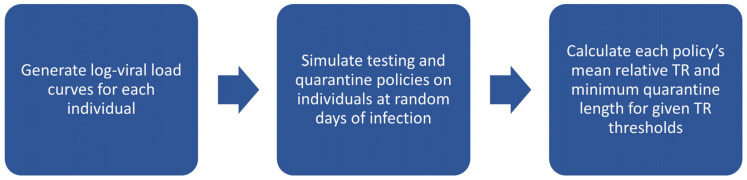
The three stages of the simulation process.

The following steps were performed for each virus variant, with 1000 iterations each consisting of 84,000 infected travellers (motivated by simulating approximately 3000 travellers for each of the 28 possible days of infection): First, individual viral load trajectories were generated for each iteration based on distributions of viral load curve parameters estimated from previous studies’ data, with travellers’ day of infection at the time of travel uniformly distributed from 1 to 28 days since infection. Then, the outcomes of quarantine and testing for each iteration were simulated for each of 462 cross-border policies (comprising 21 testing policies of varying test types and frequencies, implemented over a quarantine period of 0 to 21 days), based on viral-load-dependent test sensitivity profiles. Finally, the number of missed travellers was computed, as well as a TR estimate based on viral loads across the remaining days of infection for each traveller. This is illustrated in Scheme 1.

### 2.1. Generating Log Viral Load Curves

We include hyperparameter values for the distributions used in Document S1.

**Figure 1:**
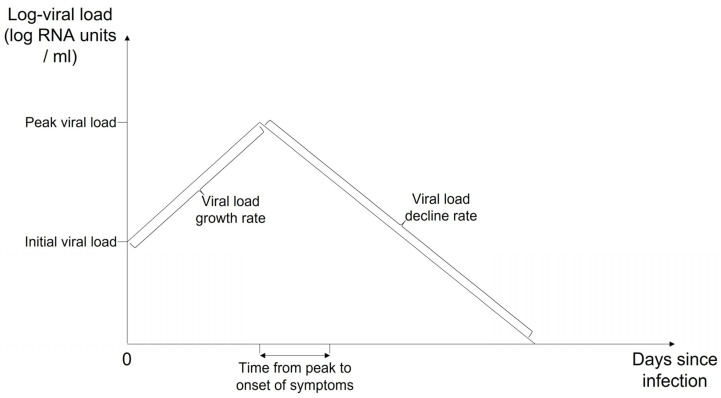
Typical log viral load curve by days since infection, labelled with parameters: peak viral load, initial viral load, viral load growth rate, viral load decline rate, time from peak to onset of symptoms.

Individual infected travellers’ log viral load curves were assumed to be piecewise-linear with an initial increasing slope followed by a decreasing slope. Curves were parametrized by initial level, growth rate (of the increasing slope), peak level and decline rate (of the decreasing slope). For symptomatic travellers, an additional parameter was the time from the peak level to the onset of symptoms. Travellers were randomly given symptomatic status at a fixed probability of 0.6 for all SARS-CoV-2 variants [14] and of 0.773 for influenza A-H1N1 [15]. A typical viral load curve is illustrated in Figure 1.

The parameter values for each individual’s log viral load curve were generated from distributions estimated with data from viral load studies. The resulting distributions of the curves’ parameters and their hyperparameter estimates are compiled in Document S1. Distributions for all the SARS-CoV-2 variants other than omicron were derived from a study of community contacts by Singanayagam et al. [16]. The hyperparameters for omicron were obtained by combining the data for the delta (vaccinated) variant with data from Young et al. [17] that compared delta and omicron viral load trajectories in vaccine breakthrough infections. The same parameter distributions were used for both symptomatic and asymptomatic travellers, in accordance with evidence that symptomatic and asymptomatic COVID-19 infections have similar viral loads [18,19]. The distribution for influenza A-H1N1 was derived from a study of experimental infections by Canini et al. [20].

Each traveller was further assigned a day of infection from 1 to 28 with equal probabilities, representing the day of their infection on which travel occurs. Travellers who had recovered from their infection on the day of travel (determined by log viral load falling below 0) were removed at this stage.

### 2.2. Simulation of Testing and Quarantine Policies

For all the virus variants, rapid antigen tests (ARTs) and polymerase chain reaction (PCR) tests were considered in application to mass testing in possible policies. PCRs were assumed to have a turnaround time of either 0, 1 or 2 days, while ARTs had a same-day turnaround. We considered 21 different types of testing policies using varying test types and frequencies, each of which could be implemented over a quarantine period of 0 to 21 days. In total, these constituted 462 cross-border policies (though with some policies being duplicates due to technicalities, as detailed in the following).

Pretests are tests performed prior to travel, so that the results are available on the day of travel. Entry tests are tests performed on the day of travel (representing testing upon arrival). Exit tests are tests performed during quarantine such that the results are available before exiting quarantine. Regular tests are tests performed at regular intervals during quarantine, such that the results are available before exiting quarantine; the first regular test is always performed on the first day of quarantine. Travellers never undergo two of the same type of test (ART or PCR) per day, though they may undergo both an ART and PCR test on the same day. Consequently, a policy in which both a PCR exit test and PCR regular test would be scheduled on the same day would be implemented as only performing one PCR test on that day, so that a similar policy without the PCR exit test would be a duplicate of it. Table 1 displays the policies that were modelled, consisting of these types of tests.

The outcomes of quarantine and testing were simulated for each policy, based on test sensitivity profiles dependent on viral load. It was assumed that symptomatic travellers whose symptoms emerged during quarantine would be captured and diagnosed. Consequently, there were three possible outcomes for travellers: diagnosed, recovered and missed. Travellers were defined to be diagnosed if any tests performed on them returned positive or symptoms were displayed. Travellers were defined to be recovered if their infection ended before they were diagnosed and before exiting quarantine. Travellers were missed if they were neither diagnosed nor recovered. Only missed travellers were able to contribute to secondary cases upon exiting quarantine.

**Figure 2:**
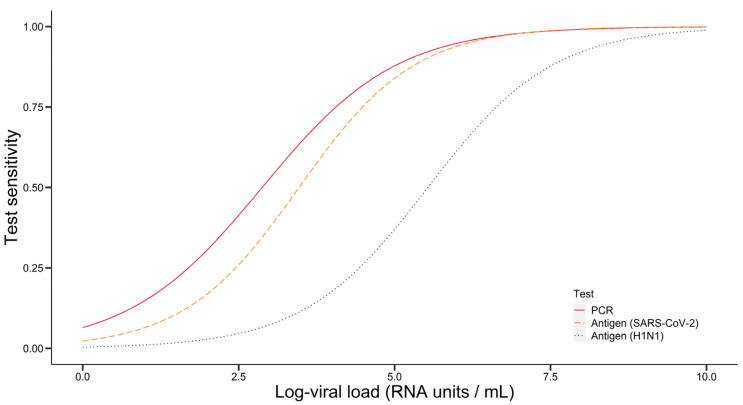
Test sensitivity by log viral load (in RNA copies/mL) for PCR (red solid), antigen for SARS-CoV-2 (orange dashed) and antigen for influenza A-H1N1 (blue dotted).

Test sensitivity profiles were obtained by fitting logistic curves to data on test sensitivity by log viral load. We note that for influenza A-H1N1, the PCR sensitivity profile was assumed to be the same as that of PCR for SARS-CoV-2, obtained from Miller et al. [21], due to a lack of influenza A-H1N1 data on PCR sensitivity by viral loads in RNA units per volume. For the influenza A-H1N1 ART, data for the QuickVue test was used [22]. For the SARS-CoV-2 ART, data for the Abbott test was used [23]. From the logistic regression of test sensitivity relative to log viral load, the coefficients yielded for each test are displayed in Table 2. The resulting curves are displayed in Figure 2, wherein test sensitivities (proportion detected) by log viral load are plotted for PCR (red), antigen for SARS-CoV-2 (orange) and antigen for influenza A-H1N1 (blue).

### 2.3. Computing Relative Total TR and Minimum Quarantine Lengths

**Figure 3:**
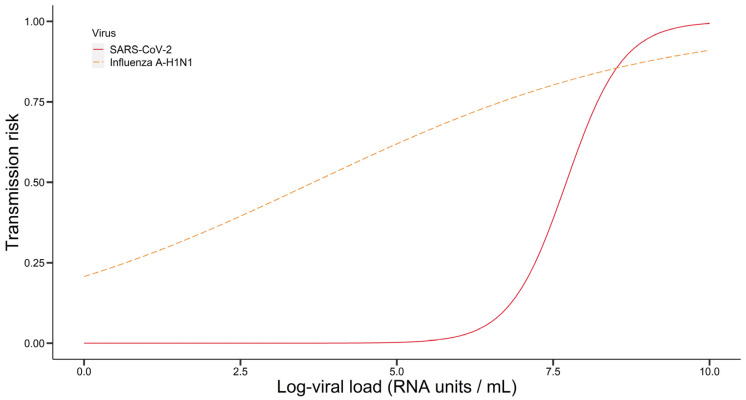
Viral transmission risk by log viral load (in RNA copies/mL) for SARS-CoV-2 (red solid) and influenza A-H1N1 (orange dashed).

For each policy, we computed a relative estimate of secondary cases due to missed infected travellers based on TR. TR denotes the probability that a given exposure event involving an infected and an uninfected individual will result in the infection of the latter. TR was computed using data on the dependence of TR on a traveller’s viral load [24,25], by fitting logistic curves to the data. The logistic regression coefficients for SARS-CoV-2 were b_0_ = −17.0 and b_1_ = 2.21, and they were b_0_ = −1.34 and b_1_ = 0.366 for influenza A-H1N1 (resulting curves in Figure 3, wherein curves for viral transmission risk by log viral load are plotted for SARS-CoV-2 and influenza A-H1N1).

The TR for SARS-CoV-2 is much lower for lower viral loads than that for influenza A-H1N1, being almost 0 until a log viral load (in RNA copies/mL) of about 6.0. It then rapidly increases, surpassing the TR for influenza A-H1N1 at a log viral load of about 8.0.

A non-normalized estimate of secondary cases for a given traveller was obtained by summing the daily TR over a traveller’s remaining days of infection, based on the assumption that the rate of occurrence of exposure events and the viral load trajectories of travellers are independent. Next, a relative estimate of the total secondary cases due to all travellers, denoted by relative total TR, was then calculated for each policy by taking the average of this aggregate over all travellers simulated and then dividing by the average obtained for the baseline policy (wherein no quarantine or testing is implemented).

We further tabulated the minimum quarantine lengths required for each strategy family to be able to suppress relative total TR below given thresholds. This was conducted for five TR thresholds: 10^−2^, 10^−3^, 10^−4^, 10^−5^ and 10^−6^ (in a ratio of expected secondary cases resulting from a given policy to that for the baseline of no quarantine or testing).

## 3. Results

### 3.1. Log Viral Load Distributions

**Figure 4:**
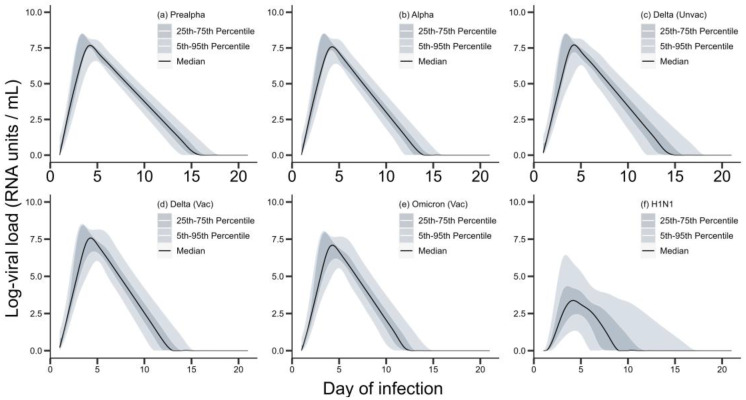
Log viral loads of virus variants by day of infection for (**a**) prealpha, (**b**) alpha, (**c**) delta (unvaccinated), (**d**) delta (vaccinated), (**e**) omicron (vaccinated) and (**f**) influenza A-H1N1. The median, 25th, 75th, 5th and 95th percentiles of log viral loads are presented by day of infection for each virus variant. All log viral loads and their rates of change are reported in log mRNA units/mL.

Figure 4 presents the simulated log viral load distributions by virus variant and day of infection.

The log viral load medians and percentiles for all the SARS-CoV-2 variants follow qualitatively similar trajectories, with relatively narrow percentile bands around a unimodal median curve, having peaks around 7.5 on day 4. The variant with the lowest median peak, omicron (vaccinated), had a peak log viral load of 6.99 (4.67, 7.76) on day 4. Delta (unvaccinated) showed the widest percentile bands among the SARS-CoV-2 variants. The percentile bands for influenza A-H1N1 are much wider, and the unimodal median curve has a much lower peak at 3.36 (1.28, 6.06) on day 4. Similar average rates of decline characterized the median curves of prealpha, alpha and delta (unvaccinated) as well as of influenza A-H1N1 with rates of 0.635, 0.683, 0.692 and 0.672 per day, respectively. Delta (vaccinated) and omicron (vaccinated) showed higher average rates of decline of 0.830 and 0.777, respectively.

The viral load trajectories of the five SARS-CoV-2 variants studied have relatively small quantitative differences. One of the main differences is in the variance in the viral load on a given day of infection, with the largest variances exhibited by delta (unvaccinated). In contrast, the viral load trajectories of influenza A-H1N1 reflected much greater variances in viral load on given days of infection. This suggests a higher frequency for influenza A-H1N1 of unusually high and unusually low viral loads relative to the median for given days, as well as a higher frequency of longer infections. Viral load trajectories for influenza A-H1N1 also had a much lower median peak than those of SARS-CoV-2 variants’ trajectories but similar rates of decline for the median curve, showing that in general influenza A-H1N1 infections tended to yield lower viral loads on given days of infection.

### 3.2. Relative Total TRs for ART-Based Strategies

**Figure 5:**
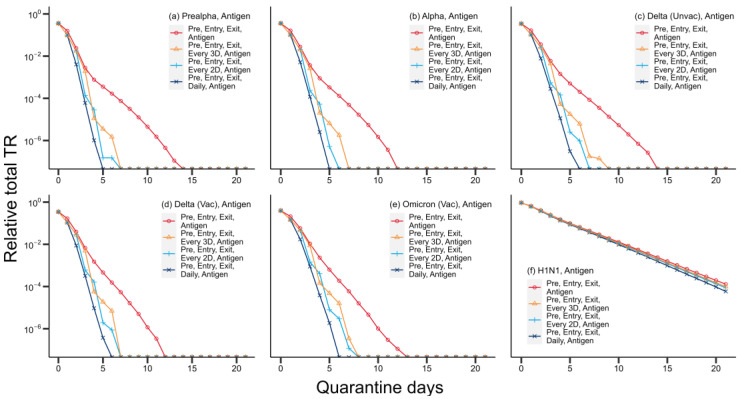
Mean relative total TR for ART-based strategies by virus variant: (**a**) prealpha, (**b**) alpha, (**c**) delta unvaccinated, (**d**) delta vaccinated, (**e**) omicron vaccinated and (**f**) influenza A-H1N1. Strategies include the following: pretesting, entry testing and exit testing (red with circles); pretesting, entry testing, testing every three days in quarantine and exit testing (orange with triangles); pretesting, entry testing, testing every two days in quarantine and exit testing (light blue with ticks); and pretesting, entry testing, testing every day in quarantine and exit testing (dark blue with crosses).

Figure 5 presents the mean relative total TR by strategy for ART-based strategies, by virus variant.

Among ART-based strategy families, more frequent regular testing yielded lower relative total TRs. For SARS-CoV-2 variants, the differences widen with longer quarantines, with sharper average rates of decline in relative total TR per quarantine day for strategy families with more frequent regular testing. The rate of decline (in relative total TR per quarantine day) for the strategy without regular in-quarantine testing (S7) ranged from 0.435 to 0.638, much slower than with daily ART testing (S11), which ranged from 1.06 to 1.96.

For influenza A-H1N1, differences between strategy families remain marginal as quarantine lengths increase. The rates of decline (in relative total TR per quarantine day) are small over the range of quarantine lengths: daily quarantine testing (S11) had a rate of 0.200, and no regular testing (S7) had 0.184.

### 3.3. Relative Total TRs for PCR-Based Strategies

**Figure 6:**
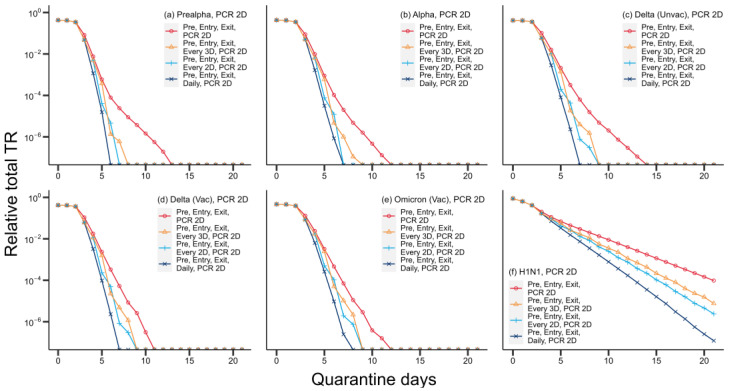
Mean relative total TR for strategies based on PCR 2D by virus variant: (**a**) prealpha, (**b**) alpha, (**c**) delta unvaccinated, (**d**) delta vaccinated, (**e**) omicron vaccinated and (**f**) influenza A-H1N1. Strategies include: pretesting, entry testing and exit testing (red with circles); pretesting, entry testing, testing every three days in quarantine and exit testing (orange with triangles); pretesting, entry testing, testing every two days in quarantine and exit testing (light blue with ticks); and pretesting, entry testing, testing every day in quarantine and exit testing (dark blue with crosses).

Figure 6 presents the mean relative total TR by strategy for strategies based on PCR 2D, by virus variant.

The PCR-based strategy families show similar patterns, with lower relative total TR from policies with more frequent regular testing. For the SARS-CoV-2 variants, differences in performance are likewise marginal with short quarantine periods but widen as quarantine periods increase. For delta (unvaccinated), the average rate of decline in relative total TR per quarantine day for the strategy with daily PCR 2D testing (S13) was 1.30, while that for no regular testing (S9) was 0.572.

For influenza A-H1N1, the differences between PCR-based strategy families grow substantially as quarantine periods increase. The rate for the strategy with daily PCR 2D testing (S13) was 0.344, while that for no regular testing (S9) was 0.191.

### 3.4. Minimum Quarantine Lengths for Given Relative TR Thresholds by Strategy

**Figure 7:**
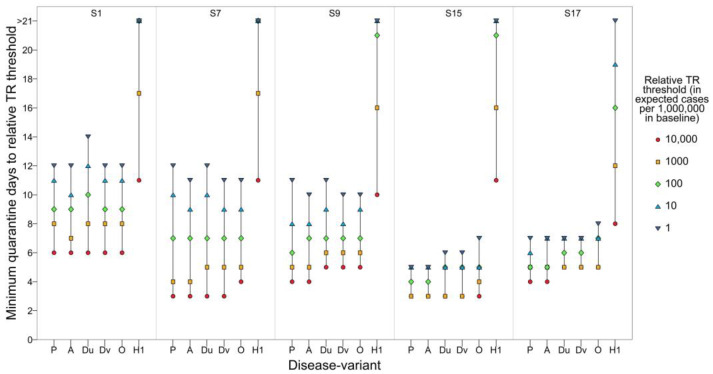
Minimum quarantine length to pass relative TR threshold, by strategy family (S1, S7, S9, S15, S17) and disease variant. P denotes prealpha, A denotes alpha, Du denotes delta (unvaccinated), Dv denotes delta (vaccinated), O denotes omicron (vaccinated) and H1 denotes influenza A-H1N1. When points overlap, the lowest (strictest) threshold is displayed rather than any higher thresholds, which require the same minimum quarantine length. The remaining strategies are presented in Table S2.1.

We tabulated minimum quarantine lengths required for relative total TR to fall below given thresholds, by disease variant and strategy family (Figure 7, Table S2.1). Thresholds are expressed as expected cases per 1,000,000 expected cases in the baseline scenario (no testing or quarantine).

For influenza A-H1N1, none of the five strategy families crossed the lowest threshold (1 in 1,000,000) within 21 days. Only S17 crossed the next-lowest threshold (10 in 1,000,000) within 21 days.

For all 21 strategy families and all thresholds, the differences between quarantine lengths needed for given SARS-CoV-2 variants were at most 2 days across SARS-CoV-2 variants for a given threshold and strategy family.

The minimum quarantine lengths for each threshold were higher across all strategy families for influenza A-H1N1 than for any SARS-CoV-2 variant. The difference between the lowest minimum quarantine length for influenza A-H1N1 and the highest minimum quarantine length for delta (unvaccinated) increased from 2 days at the highest threshold to more than 7 days for the lowest threshold. This tendency for the differences between SARS-CoV-2 variants and H1N1 to grow larger for stricter thresholds held across SARS-CoV-2 variants (Table S2.1).

The differences between the minimum quarantine lengths required for strategies with no regular testing (S1 to S9) across SARS-CoV-2 variants were small for each threshold, ranging from 3 to 6 days for the highest threshold and 10 to 14 days for the lowest. The minimum quarantine lengths for strategy families with regular testing (S10 to S21) ranged from 2 to 5 days for the highest threshold and 4 to 9 days for the lowest.

For influenza A-H1N1, there were small differences between all strategies with no regular testing or regular ART testing: minimum quarantine lengths were 10 or 11 days for the highest threshold and more than 21 days for the lowest two. Minimum quarantine lengths for strategy families with regular PCR testing were 7 to 9 days for the highest threshold and 16 to 21 days for the second-lowest. Only strategies with daily PCR testing could attain the lowest threshold within a 21-day quarantine, with minimum lengths of 18 or 19 days.

For SARS-CoV-2, at any testing frequency, SARS-CoV-2 variant and threshold, the minimum quarantine length for PCR 0D (0-day turnaround) did not exceed ART’s, ART’s did not exceed PCR 1D (1-day turnaround)’s, and PCR 1D’s did not exceed PCR 2D’s. The difference between PCR 0D and PCR 2D was at most 2 days for any testing frequency, SARS-CoV-2 variant and threshold.

For influenza A-H1N1, at any given testing frequency and threshold, PCR 0D’s required minimum quarantine length did not exceed PCR 1D’s, PCR 1D’s did not exceed PCR 2D’s, and PCR 2D’s did not exceed ART’s. Differences between strategy families grew with lower thresholds and higher testing frequencies, with a range of 3 days at the highest threshold and testing every 3 days and a range of at least 6 days at the second-lowest threshold and daily testing.

## 4. Discussion

The results indicate that for viruses with high typical viral loads and minimal TR at low viral loads (such as SARS-CoV-2) it suffices to use tests with lower sensitivity (such as ARTs) and to have shorter quarantine periods to suppress TR to low levels. Conversely, for viruses with lower typical viral loads and moderate TR even at low viral loads (such as influenza A-H1N1), tests with high sensitivity (such as PCR) and long quarantine periods are necessary to attain low levels of TR.

Relatively low viral loads, coupled with ARTs low sensitivity for influenza A-H1N1 across all but the highest viral loads, resulted in regular ART testing barely decreasing quarantine lengths needed to suppress transmission to desired rates. Thus, regular ART testing is a poor substitute for time in quarantine for influenza A-H1N1, because of cases whose viral loads are too low to be diagnosed reliably by ART (based on influenza A-H1N1′s ART sensitivity data), as studies during the 2009 pandemic have also found [26,27].

In contrast, ARTs performed well for the SARS-CoV-2 variants, in agreement with empirical sensitivity studies [28,29]. For SARS-CoV-2, the median peak log viral load was high, and ARTs sensitivity was almost comparable to PCR’s at typical viral loads, yielding a much steeper decline in relative total TR under regular ART testing than without, across SARS-CoV-2 variants.

For influenza A-H1N1, PCR had sufficiently high sensitivity that PCR testing could significantly reduce quarantine lengths required to suppress TR to given thresholds, but it remains likely that a few influenza A-H1N1 cases with particularly low viral loads are missed even with long quarantines and regular PCR testing every few days. Further, lower viral loads still carry significant TR for influenza A-H1N1. We note that the PCR sensitivity data used was that for SARS-CoV-2, having assumed that PCR’s sensitivity for both viruses is similar. Evidence strongly supports PCR’s high sensitivity for influenza A-H1N1 among symptomatic and hospitalized patients, whose viral loads tend to be higher [30], but particularly low viral loads may go undetected [31], as may be more common in asymptomatic cases.

PCR yielded even better performance for SARS-CoV-2 variants, in agreement with empirical sensitivity studies [32]. The strategy family with regular PCR 2D testing every 3 days (S21) passed the lowest threshold within a 9-day quarantine length for all SARS-CoV-2 variants, demonstrating PCR’s high sensitivity for SARS-CoV-2 variants and also the low TR associated with lower viral loads for SARS-CoV-2.

Compared to SARS-CoV-2 variants, influenza A-H1N1 required longer minimum quarantine lengths for given TR thresholds across strategy families, with the differences growing with stricter thresholds. For viruses which, like influenza A-H1N1, have low typical viral load ranges and moderate TR even at low viral loads, long quarantines are likely necessary to suppress TR below strict thresholds, regardless of testing strategy. Conversely, viruses which, like SARS-CoV-2, have high typical viral loads (that is, at which available tests exhibit good sensitivity) and low TR at low viral loads are likely effectively suppressed by testing, although moderate quarantine periods are necessary for stricter TR thresholds.

For SARS-CoV-2 variants, regular ART testing slightly outperformed regular PCR testing (with 1- or 2-day delays). This reflects a trade-off between testing delay and test sensitivity. A delay of N days precludes testing up to N-1 days before the last day of quarantine (assuming results must be available by the last day), countervailing the additional sensitivity offered by such tests. Tests with shorter or no delay can be administered during this period and are well-timed to capture cases wherein viral load peaks near the end of quarantine. This concurs with previous findings on the primacy of testing frequency and delay in strategies’ performance for suppressing COVID-19 transmission [33]. For influenza A-H1N1, this effect could not supersede the superior sensitivity of PCR testing, as the difference in PCR’s sensitivity compared to ARTs for influenza A-H1N1 was much larger than for SARS-CoV-2.

We additionally found that SARS-CoV-2 variants did not vary sufficiently in terms of viral load trajectories for significant variation in the pattern of outcomes. The high sensitivities of available tests relative to typical viral loads made it feasible to perform regular testing, even with ARTs, in lieu of longer quarantine periods, to minimize imported and resulting secondary cases. Further, the incidence of cases with exceptionally low viral loads (and which were thus difficult to diagnose with tests) posed minimal TR, which also made testing advantageous.

Influenza A-H1N1 provided an instructive contrast to SARS-CoV-2. The low typical viral loads for influenza A-H1N1 (relative to available tests’ sensitivities) made ARTs relatively ineffective at diagnosis, so that regular in-quarantine ART testing could not significantly reduce the quarantine period needed for the highest TR threshold (reducing the minimum quarantine period by just 1 day in comparison to no regular in-quarantine testing). This was exacerbated by the significant TR associated with low viral loads for influenza A-H1N1, which made ART testing an ineffective alternative to time in quarantine. Regular PCR testing allowed for significant reductions in quarantine lengths while attaining higher thresholds but remained insufficient to attain the lowest thresholds without long quarantine periods.

We remark that the effect of incoming travellers on the transmission dynamics of the country as a whole depends on the ratio of infected travellers to the susceptible, infected and immune (recovered or vaccinated) populations, as well as other factors determining the reproduction number and equilibrium infection level. We do not discuss this effect in detail as these quantities are dependent on the situation in a given country. However, we note that from the perspective of such SIR-type models, infected travellers can be viewed as increasing the infected population in a country at a given point in time. Correspondingly, border control policies have the effect of scaling this increase by a factor that is equal to the mean relative TR of a given policy.

We note certain limitations of the model. Firstly, it assumes that test outcomes depend solely on viral loads on the testing day, and for SARS-CoV-2, the same sensitivity curves were used across variants. This omits other possible factors, such as test administrators’ competence. However, experimental evidence suggests similar test sensitivities across SARS-CoV-2 variants [34,35]. Secondly, the use of test sensitivity data available for specific manufacturers’ tests may limit our findings’ validity. Replicating the simulations with alternative manufacturers’ data will allow corroboration of the conclusions about the disease-specific efficacy of ARTs and PCR.

Third, all symptomatic travellers are assumed isolated until recovery when symptoms emerge in quarantine. Realistically, this will depend on symptom severity and the availability of isolation facilities. Fourth, full adherence to testing and quarantine requirements is assumed. In real-world implementations, this depends on the strictness of enforcement, and non-adherence may correlate with TR through risky behaviours.

Fifth, elaboration of within-host dynamics would help to corroborate the statistical models of viral load kinetics used in this study. Sixth, the TR curves used depend solely on viral load, omitting behavioural factors, such as demographic and temporal variation in travellers’ behaviour, local regulations and biological factors, such as immune evasion, which is believed to increase transmissibility for certain SARS-CoV-2 variants [36]. In particular, symptomatic and asymptomatic travellers may pose different transmission risks due to different frequencies of exposure events. Symptomatic travellers may self-quarantine upon symptom emergence or, conversely, infect others more readily through their symptoms.

Finally, TR may not reflect the risk of severe illness, which is of particular interest for countries seeking to transition to controlled endemic states. However, this likely depends on the receiving country’s demographics, such as vaccination rates across age groups.

Viral load and TR profiles of emerging diseases are important factors determining testing effectiveness and quarantine lengths needed with given testing regimens to suppress secondary cases from infected travellers. Data on these parameters will be key to formulating border-control strategies for future disease outbreaks that are cost-effective and minimally prohibitive to travellers, while being sufficiently rigorous for given risk targets.

Further simulation studies in this vein may systematically vary parameters defining a disease’s viral load and TR profiles, as well as a hypothetical test’s sensitivity profile, to determine more comprehensively how these parameters affect the effectiveness of quarantine and testing policies.

## Figures and Tables

**Table 1:** Policies modelled. PCR 0D denotes a PCR test with a 0-day turnaround time, PCR 1D denotes a 1-day turnaround time and PCR 2D denotes a 2-day turnaround time.

Strategy Number	Pretest Type	Entry Test Type	Exit Test Type	Regular Test Type	Days Per Regular Test
S1	-	-	-	-	-
S2	PCR 0D	ART	-	-	-
S3	ART	ART	-	-	-
S4	PCR 1D	ART	-	-	-
S5	PCR 2D	ART	-	-	-
S6	PCR 0D	ART	PCR 0D	-	-
S7	ART	ART	ART	-	-
S8	PCR 1D	ART	PCR 1D	-	-
S9	PCR 2D	ART	PCR 2D	-	-
S10	PCR 0D	ART	PCR 0D	PCR 0D	1
S11	ART	ART	ART	ART	1
S12	PCR 1D	ART	PCR 1D	PCR 1D	1
S13	PCR 2D	ART	PCR 2D	PCR 2D	1
S14	PCR 0D	ART	PCR 0D	PCR 0D	2
S15	ART	ART	ART	ART	2
S16	PCR 1D	ART	PCR 1D	PCR 1D	2
S17	PCR 2D	ART	PCR 2D	PCR 2D	2
S18	PCR 0D	ART	PCR 0D	PCR 0D	3
S19	ART	ART	ART	ART	3
S20	PCR 1D	ART	PCR 1D	PCR 1D	3
S21	PCR 2D	ART	PCR 2D	PCR 2D	3

**Table 2:** Logistic regression coefficients for test sensitivities against log viral load (in RNA copies/mL).

Test Type	b_0_ (Intercept)	b_1_ (Log Viral Load)
PCR	−2.67	0.929
ART (COVID-19)	−3.75	1.08
ART (influenza A-H1N1)	−5.53	1.00

## Data Availability

The data presented in this study are available in Tables S1.1 to S1.6 and S2.1. The R (version 4.2.2) code that implements the model and all required parameter files are available at https://github.com/nigellwh/border-control (accessed on 12 April 2023).

## Supplementary Materials

The following supporting information can be downloaded at: https://www.mdpi.com/article/10.3390/v15040978/s1, Document S1: Viral load curve parameter distributions and hyperparameters; Document S2: Variable descriptions for Tables S1.1–S1.6; Document S3—Variable descriptions for Table S2.1; Table S1.1—Outcomes for Prealpha; Table S1.2—Outcomes for Alpha; Table S1.3—Outcomes for Delta (Unvac); Table S1.4—Outcomes for Delta (Vac); Table S1.5—Outcomes for Omicron (Vac); Table S1.6—Outcomes for Influenza A H1N1; Table S2.1—Min Quarantine Lengths.
