# Supplementary material for: Border Control for Infectious Respiratory Disease Pandemics: A Modelling Study for H1N1 and Four Strains of SARS-CoV-2
Source: Viruses. 2023 Apr 16;15(4):978. doi: 10.3390/v15040978 (PMC10144227; doi:10.3390/v15040978)
Supplement: Supplementary file 1 [file viruses-15-00978-s001.zip › Document S1 - Viral load curve parameter distributions and hyperparameters.pdf]

# Document S1. Viral load curve parameter distributions and hyperparameters

## Table S1. Distributions and means of log-viral load curve parameters' underlying normal variables

| Disease-variant | Asymptomatic / symptomatic | Parameter | Distribution | Mean (if log-normal, mean of log) |
|---|---|---|---|---|
| COVID-19 prealpha | Both | Initial log-viral load | Constant | 1.49 |
| | | Rate of increase in log-viral load | Log-normal | 1.18 |
| | | Peak log-viral load | Normal | 8.14 |
| | | Rate of decrease in log-viral load | Normal | 0.69 |
| | Symptomatic | Days from peak to onset of symptoms | Normal | 0.6 |
| COVID-19 alpha | Both | Initial log-viral load | Constant | 1.49 |
| | | Rate of increase in log-viral load | Log-normal | 1.14 |
| | | Peak log-viral load | Normal | 8.14 |
| | | Rate of decrease in log-viral load | Normal | 0.82 |
| | Symptomatic | Days from peak to onset of symptoms | Normal | 0.6 |
| COVID-19 delta (unvaccinated) | Both | Initial log-viral load | Constant | 1.49 |
| | | Rate of increase in log-viral load | Log-normal | 1.03 |
| | | Peak log-viral load | Normal | 8.14 |
| | | Rate of decrease in log-viral load | Normal | 0.79 |
| | Symptomatic | Days from peak to onset of symptoms | Normal | 0.6 |
| COVID-19 delta (vaccinated) | Both | Initial log-viral load | Constant | 1.49 |
| | | Rate of increase in log-viral load | Log-normal | 0.99 |
| | | Peak log-viral load | Normal | 8.14 |
| | | Rate of decrease in log-viral load | Normal | 0.95 |
| | Symptomatic | Days from peak to onset of symptoms | Normal | 0.6 |
| COVID-19 omicron (vaccinated) | Both | Initial log-viral load | Constant | 1.0084 |

| | | | | |
|---|---|---|---|---|
| | | Rate of increase in log-viral load | Log-normal | 0.99 |
| | | Peak log-viral load | Normal | 7.6584 |
| | | Rate of decrease in log-viral load | Normal | 0.95 |
| | Symptomatic | Days from peak to onset of symptoms | Normal | 0.6 |
| Influenza A-H1N1 | Asymptomatic | Initial log-viral load | Constant | 0 |
| | | Rate of increase in log-viral load | Log-normal | -0.30231 |
| | | Peak log-viral load | Normal | 1.72 |
| | | Rate of decrease in log-viral load | Log-normal | -0.41705 |
| | Symptomatic | Initial log-viral load | Constant | 0 |
| | | Rate of increase in log-viral load | Log-normal | 0.69197 |
| | | Peak log-viral load | Normal | 4.60588 |
| | | Rate of decrease in log-viral load | Log-normal | 0.18735 |
| | | Days from peak to onset of symptoms | Normal | -0.07353 |

**Table S2. Covariance matrix of log-viral load curve parameters' underlying normal variables for COVID-19 prealpha**

| | Initial log-viral load | Rate of increase in log-viral load | Peak log-viral load | Rate of decrease in log-viral load | Days from peak to onset of symptoms (symptomatic only) |
|---|---|---|---|---|---|
| Initial log-viral load | 0 | 0 | 0 | 0 | 0 |
| Rate of increase in log-viral load | 0 | 0.09090 | 0.01166 | -0.00342 | 0 |
| Peak log-viral load | 0 | 0.01166 | 0.0081 | -0.00238 | 0 |
| Rate of decrease in log-viral load | 0 | -0.00342 | -0.00238 | 0.0036 | 0 |
| Days from peak to onset of symptoms (symptomatic only) | 0 | 0 | 0 | 0 | 0.0225 |

**Table S3. Covariance matrix of log-viral load curve parameters' underlying normal variables for COVID-19 alpha**

| | Initial log-viral load | Rate of increase in log-viral load | Peak log-viral load | Rate of decrease in log-viral load | Days from peak to onset of symptoms (symptomatic only) |
|---|---|---|---|---|---|
| Initial log-viral load | 0 | 0 | 0 | 0 | 0 |
| Rate of increase in log-viral load | 0 | 0.1024 | 0.01241 | -0.00455 | 0 |
| Peak log-viral load | 0 | 0.01241 | 0.0081 | -0.00297 | 0 |
| Rate of decrease in log-viral load | 0 | -0.00455 | -0.00297 | 0.00563 | 0 |
| Days from peak to onset of symptoms (symptomatic only) | 0 | 0 | 0 | 0 | 0.0225 |

**Table S4. Covariance matrix of log-viral load curve parameters' underlying normal variables for COVID-19 delta (unvaccinated)**

| | Initial log-viral load | Rate of increase in log-viral load | Peak log-viral load | Rate of decrease in log-viral load | Days from peak to onset of symptoms (symptomatic only) |
|---|---|---|---|---|---|
| Initial log-viral load | 0 | 0 | 0 | 0 | 0 |
| Rate of increase in log-viral load | 0 | 0.11223 | 0.01303 | -0.00796 | 0 |
| Peak log-viral load | 0 | 0.01303 | 0.0081 | -0.00495 | 0 |
| Rate of decrease in log-viral load | 0 | -0.00796 | -0.00495 | 0.01563 | 0 |
| Days from peak to onset of symptoms (symptomatic only) | 0 | 0 | 0 | 0 | 0.0225 |

**Table S5. Covariance matrix of log-viral load curve parameters' underlying normal variables for COVID-19 delta (vaccinated) and omicron (vaccinated)**

| | Initial log-viral load | Rate of increase in log-viral load | Peak log-viral load | Rate of decrease in log-viral load | Days from peak to onset of symptoms (symptomatic only) |
|---|---|---|---|---|---|
| Initial log-viral load | 0 | 0 | 0 | 0 | 0 |
| Rate of increase in log-viral load | 0 | 0.10563 | 0.01262 | -0.00709 | 0 |
| Peak log-viral load | 0 | 0.01262 | 0.0081 | -0.00455 | 0 |
| Rate of decrease in log-viral load | 0 | -0.00709 | -0.00455 | 0.01323 | 0 |
| Days from peak to onset of symptoms (symptomatic only) | 0 | 0 | 0 | 0 | 0.0225 |

**Table S6. Covariance matrix of log-viral load curve parameters' underlying normal variables for influenza A-H1N1 (asymptomatic)**

| | Initial log-viral load | Rate of increase in log-viral load | Peak log-viral load | Rate of decrease in log-viral load |
|---|---|---|---|---|
| Initial log-viral load | 0 | 0 | 0 | 0 |
| Rate of increase in log-viral load | 0 | 0.14266 | -0.03314 | 0.10667 |
| Peak log-viral load | 0 | -0.03314 | 0.45511 | -0.08302 |
| Rate of decrease in log-viral load | 0 | 0.10667 | -0.08302 | 0.10516 |

**Table S7. Covariance matrix of log-viral load curve parameters' underlying normal variables for influenza A-H1N1 (symptomatic)**

| | Initial log-viral load | Rate of increase in log-viral load | Peak log-viral load | Rate of decrease in log-viral load | Days from peak to onset of symptoms |
|---|---|---|---|---|---|
| Initial log-viral load | 0 | 0 | 0 | 0 | 0 |
| Rate of increase in log-viral load | 0 | 0.27304 | 0.48881 | 0.08925 | -0.00734 |
| Peak log-viral load | 0 | 0.48881 | 2.25512 | 0.48155 | 0.07166 |
| Rate of decrease in log-viral load | 0 | 0.08925 | 0.48155 | 0.29023 | 0.04914 |
| Days from peak to onset of symptoms | 0 | -0.00734 | 0.07166 | 0.04914 | 0.66867 |
